# Involuntary Urine Loss in Menopause—A Narrative Review

**DOI:** 10.3390/jcm14217664

**Published:** 2025-10-29

**Authors:** Lucian Șerbănescu, Sebastian Mirea, Paris Ionescu, Laura Andra Petrica, Ionut Ciprian Iorga, Monica Surdu, Traian Virgiliu Surdu, Vadym Rotar

**Affiliations:** 1 Faculty of Medicine, Ovidius University of Constanta, 900470 Constanta, Romania; paris.ionescu@365.univ-ovidius.ro (P.I.); laurapetrica@yahoo.com (L.A.P.); ionut.iorga@365.univ-ovidius.ro (I.C.I.); monica@surdu.ro (M.S.); traian@surdu.ro (T.V.S.); rotarvadym.obst.gyn@gmail.com (V.R.); 2 County Clinical Emergency Hospital “Sf. Ap. Andrei”, 900591 Constanta, Romania; sebastian.mirea@umft.ro; 3 Techirghiol Balneal and Rehabilitation Sanatorium, 906100 Constanta, Romania

**Keywords:** menopause, balneotherapy, stress urinary incontinence, pelvic floor muscle, kegel exercises, vaginal surgery

## Abstract

**Objective**: This narrative review aims to summarize current evidence on the epidemiology, risk factors, clinical patterns, and therapeutic strategies for urinary incontinence in menopausal women. **Background**: Urinary incontinence (UI) is a common, multifactorial condition that disproportionately affects women, with prevalence rising during pregnancy and post menopause. While stress urinary incontinence (SUI) predominates in younger and early postmenopausal women, urgency urinary incontinence (UUI) and mixed urinary incontinence (MUI) become increasingly prevalent with age and duration following menopause. Additional determinants, such as obesity, comorbidities, polypharmacy, and menopausal symptoms, burden further contribute to symptom severity and persistence. **Materials and Methods**: For the materials and methods, we used over 150 specialized studies and meta-analyses published in the specialized literature on this subject, of which 99 are mentioned in the bibliography of this narrative review. These materials are some of the most significant and up-to-date that address this complex topic. **Content**: This narrative review discusses the impact of menopause-related hormonal decline on the genitourinary tract, highlighting the role of estrogen deficiency in genitourinary syndrome of menopause (GSM). It addresses distinct patterns of UI across life stages, including pregnancy and the early and late postmenopause periods, and explores modifiable risk factors such as body mass index. Specific attention is given to nocturnal incontinence, medication-related effects, and coital incontinence, which significantly impair sexual health and quality of life. Therapeutic strategies are presented in a stepwise manner: conservative measures (pelvic floor muscle training), hormonal approaches (local vaginal estrogen), combination regimens, and surgical interventions, including midurethral slings, colposuspension, bulking agents, and neuromodulatory techniques. **Future perspectives**: Emerging modalities such as balneotherapy and energy-based therapies show promise but require further investigation. **Conclusions**: Urinary incontinence in menopausal women is best addressed through an individualized, multimodal approach that integrates conservative, hormonal, minimally invasive, and surgical options. Combination therapies demonstrate superior efficacy in addressing both continence and GSM-related symptoms. Future research should refine patient selection, optimize multimodal treatment algorithms, and prioritize long-term outcomes and quality-of-life measures in clinical decision-making.

## 1. Background

Menopause is defined as the permanent cessation of menstruation, resulting from the depletion of ovarian follicular activity and the consequent decline in estrogen production. The clinical diagnosis is established retrospectively after 12 consecutive months of amenorrhea, in the absence of other physiological or pathological causes [1,2]. The mean age of natural menopause is approximately 50–51 years, with variations influenced by individual characteristics and population factors [1,2,3,4].

Menopause marks the end of reproductive capacity and is associated with profound endocrine changes, most notably a substantial reduction in circulating estradiol and progesterone levels, accompanied by a compensatory rise in follicle-stimulating hormone (FSH) and luteinizing hormone (LH) [5,6]. The transitional phase, referred to as perimenopause, is characterized by irregular menstrual cycles and significant hormonal fluctuations, frequently accompanied by vasomotor symptoms (hot flashes, night sweats), sleep disturbances, mood alterations, and a wide spectrum of genitourinary manifestations [1,7,8,9].

Premature menopause is defined as menopause occurring before the age of 40, whereas early menopause refers to its onset between the ages of 40 and 45. Surgical menopause describes the abrupt cessation of ovarian function following bilateral oophorectomy performed before the natural menopausal age [1,3].

According to the American Heart Association, menopause constitutes a critical stage in a woman’s life, with major implications for long-term cardiovascular and metabolic health [1]. Importantly, most women will spend up to 40% of their lifespan in the postmenopausal state, highlighting the considerable clinical and socio-economic relevance of this transition [1,4].

Urinary incontinence is defined as the involuntary loss of urine, representing a common and often distressing medical condition that can significantly impact quality of life. It occurs when the control over the urinary sphincter is weakened or lost, leading to the unintentional leakage of urine. This condition can affect individuals of all ages but is more prevalent among women, particularly during or after pregnancy, menopause, or advanced age. Urinary incontinence is not a disease in itself, but rather a symptom of various underlying conditions affecting the urinary tract, pelvic floor muscles, or nervous system. The etiology is multifactorial, and the condition may manifest in different forms, including stress incontinence, urge incontinence, overflow incontinence, functional incontinence, or mixed types. Effective diagnosis and management require a thorough understanding of its causes, risk factors, and psychosocial implications.

Urinary incontinence is significantly more prevalent in women than in men across all age groups. Among community-dwelling adults, the prevalence in women ranges from 12% to 55% depending on age and definition, while in men it is lower, typically 3% to 34% in older adults, with the female-to-male ratio being 1.3–2.0 in older adults and 4.1–4.5 in younger and middle-aged adults [10,11,12].

The type of incontinence also differs by gender. In women, stress urinary incontinence (SUI)—involuntary leakage with increased intra-abdominal pressure—is the most common subtype in younger and middle-aged women, accounting for up to two-thirds of cases. With advancing age, urgency and mixed incontinence become more prevalent, so that in women over 65, urge and mixed types predominate. In men, urge incontinence and overflow incontinence are more common, often related to prostatic disease or neurologic conditions, and stress incontinence is rare except after prostate surgery [11,12,13].

The risk factors for urinary incontinence also differ by gender. In women, pregnancy, vaginal delivery, parity, menopause, and obesity are major contributors, while in men, lower urinary tract obstruction and neurologic disease are more prominent. The impact on quality of life is substantial in both sexes, but women are more likely to report symptoms and seek care [14,15,16].

Urinary incontinence affects 41–52% of pregnant women, with stress urinary incontinence (SUI) being the most prevalent subtype due to pelvic floor muscle weakening from hormonal shifts, increased intra-abdominal pressure, and prior vaginal delivery. Urgency urinary incontinence (UUI) and mixed forms are also reported, with overactive bladder symptoms being present in up to 65% of cases. Risk factors include previous incontinence, vaginal birth history, higher body mass index (BMI), older maternal age, and pelvic floor dysfunction. Despite its impact on quality of life, help-seeking rates remain low [17,18,19,20,21,22,23].

In menopausal women, urinary incontinence is equally common, affecting 45–50%. While SUI is more frequent before menopause, UUI and mixed incontinence dominate postmenopause, largely due to estrogen deficiency, reduced urethral pressure, and genitourinary atrophy. These symptoms, part of the genitourinary syndrome of menopause, include urgency, frequency, nocturia, and recurrent infections. A strong link exists between overall menopausal symptom burden and urinary incontinence severity. In both populations, incontinence remains underdiagnosed and undertreated, despite its physical, emotional, and social consequences [14,24,25,26,27].

## 2. Materials and Methods

As materials and methods, we used over 150 specialized studies and meta-analyses published in the specialized literature on this subject, of which 97 are mentioned in the bibliography of this narrative review. These materials are some of the most significant and up-to-date that address this complex topic.

## 3. Content

### 3.1. The Effects of Menopause on the Genitourinary Tract

The genital and urinary changes occurring during menopause are collectively termed the genitourinary syndrome of menopause (GSM). These are primarily attributed to estrogen deficiency and encompass the following:

- Vaginal dryness, burning, and irritation—due to thinning, loss of elasticity, and increased fragility of the vaginal epithelium, leading to reduced lubrication and greater susceptibility to trauma and irritation.
- Dyspareunia—resulting from thinning and reduced elasticity of the vaginal walls, compounded by decreased lubrication.
- Vulvar atrophy—characterized by loss of labial and vulvar fullness, narrowing of the introitus, and pale or inflamed mucosal surfaces.
- Vaginal pruritus and bleeding—secondary to the fragility of atrophic tissue, which bleeds easily, especially after minor trauma or intercourse.
- Urinary symptoms—including urgency, frequency, nocturia, dysuria, and increased susceptibility to recurrent urinary tract infections, often related to atrophic changes in the urethra and bladder trigone.
- Urinary incontinence—with both stress and urge incontinence becoming more prevalent or exacerbated during menopause.

These symptoms are progressive, significantly impair quality of life, and are frequently underdiagnosed and undertreated. Consequently, clinicians are encouraged to proactively assess the presence of GSM in menopausal women [28,29,30,31,32,33,34,35,36,37].

Since the subject of our descriptive review is primarily focused on the menopausal period, we allow ourselves to make a few observations about another important period in a woman’s life, namely pregnancy. It is well known that both menopause and pregnancy are accompanied by significant changes in the female hormonal constellation—menopause through the abrupt decline in the main steroid hormones, and pregnancy through a substantial increase in them.

During the pregnancy, the entire uterine ligamentous apparatus undergoes changes induced both by the extensive increase in the volume of the uterus and by hormonal modifications. These changes can have a significant impact throughout a woman’s life.

Although, theoretically, it would be odd to draw an analogy between involuntary urine loss in menopausal women and in pregnant women, we nevertheless feel compelled to present some brief information about this condition in pregnant women as well.

The prevalence of urinary incontinence (UI) in pregnant women increases with advancing gestation, with the lowest rates in the first trimester and the highest in the third trimester. Large systematic reviews and cohort studies consistently report this pattern. For example, pooled data indicate that UI prevalence is approximately 4–19% in the first trimester, 23–40% in the third trimester, and overall rates during pregnancy range from 35 to 58% depending on population and methodology. One large Norwegian cohort found that UI prevalence increased from 26% pre-pregnancy to 58% by week 30 of gestation. Another meta-analysis reported a third-trimester prevalence of 32%, while a prospective Italian cohort found 4.4% in the first trimester and 23.7% in the third [38,39,40,41].

Stress urinary incontinence (SUI) is the most common type during pregnancy, accounting for 48–63% of cases, with urge incontinence and mixed types being less frequent. Most women report mild to moderate leakage, typically in small amounts and less than once per week. The impact on quality of life is generally mild to moderate, but increases with severity and later gestational age [20,23,42].

Risk factors for UI in pregnancy include higher parity, advanced maternal age, higher BMI, prior vaginal delivery, and a history of UI before pregnancy. The majority of women do not seek care, because symptoms lead to minimal bother or belief that symptoms will resolve postpartum [43,44].

In pregnancy, urinary incontinence is a frequent and predominantly stress-related issue that becomes more prevalent with advancing gestational age and, despite being usually mild, it can significantly affect quality of life [19,38,45].

In general, these changes are reversible after the pregnancy period ends. Although numerous studies indicate that the number of a woman’s births is directly related to the incidence and severity of involuntary urine loss, in our opinion, a much more important role is played by complications that occur during childbirth (vaginal, cervical or urethral tears, muscle fiber dehiscence, etc.) [22], as well as the absence of pelvic muscle recovery exercises (Kegel exercises), both during the postpartum period and afterwards [46].

A woman’s obstetric history often plays an important role in cases of urinary incontinence during menopause. An important correlation between urinary incontinence during pregnancy and in menopause is observed by some specialized studies, which conclude that the strongest risk factor for incontinence later in life is incontinence during pregnancy [47].

### 3.2. Urinary Incontinence and Menopause

#### 3.2.1. Influence of Age and Time Since Menopause

The distribution of urinary incontinence in postmenopausal women according to the time elapsed since the onset of menopause is characterized by a shift in the predominant type and a gradual increase in overall prevalence with advancing age, rather than a sharp change at menopause itself.

Stress urinary incontinence (SUI) is more common in premenopausal and early postmenopausal women, while urgency urinary incontinence (UUI) and mixed urinary incontinence (MUI) become more prevalent over time, since menopause progresses. There is no evidence for a specific increase in the overall prevalence of urinary incontinence immediately at the onset of menopause; rather, the prevalence increases in a linear fashion with age. Over time, the proportion of women with UUI and MUI rises, particularly in those more than a decade past menopause, while SUI becomes less dominant [13,26,48].

Urodynamic studies confirm that SUI is the predominant type in premenopausal women, but after menopause, the prevalence of SUI with concomitant detrusor overactivity (i.e., mixed incontinence) increases steadily, becoming the most common type in women over 66 years old [48]. Cross-sectional and longitudinal data also show a weak but statistically significant positive correlation between duration of menopause and urinary incontinence symptoms, but this association is modest compared to the effect of age and other risk factors, such as parity and weight gain [49,50].

The prevalence of urinary incontinence in postmenopausal women increases gradually with time since menopause, with a shift from stress to urgency and mixed incontinence subtypes as women age. The time elapsed since menopause is less influential than chronological age and other risk factors in determining the distribution of incontinence types [13,26,49].

Women aged 60 years and older are most affected by urinary incontinence among postmenopausal women. In women aged 40–59 years, stress urinary incontinence (SUI) is most common, while in those aged 60 years and older, urgency urinary incontinence (UUI) and mixed urinary incontinence (MUI) become increasingly prevalent and eventually predominate [13,16,48].

The prevalence of SUI is highest in the early postmenopausal years and then declines, whereas UUI and MUI rise steadily with advancing age and longer duration since menopause. Urodynamic data confirm that SUI with concomitant detrusor overactivity (i.e., MUI) becomes the predominant type after age 66, reflecting the cumulative effects of aging and time since menopause [48]. There is a weak but statistically significant positive correlation between duration of menopause and urinary incontinence symptoms, but this is less influential than chronological age [49].

The highest prevalence and greatest burden of urinary incontinence in postmenopausal women occur in those aged 60 years and older, with a shift from stress to urgency and mixed incontinence as time since menopause increases [13,16,48,49].

#### 3.2.2. Role of Body Mass Index (BMI) and Obesity

The distribution of urinary incontinence (UI) in postmenopausal women increases progressively with a higher body mass index (BMI). The prevalence and severity of UI are significantly higher in women with overweight (BMI 25–29.9 kg/m^2^) and obesity (BMI ≥ 30 kg/m^2^) compared to those with normal BMI. For example, pooled analyses show that overweight postmenopausal women have an odds ratio (OR) of 1.27 (95% CI: 1.17–1.37) for UI, while those with obesity have an OR of 1.60 (95% CI: 1.42–1.81), and those with BMI ≥ 35 kg/m^2^ have an OR of 1.85 (95% CI: 1.59–2.16), compared to women with normal BMI [51].

The relationship is approximately linear, with each 5 kg/m^2^ increase in BMI associated with a 20% higher risk of UI (summary relative risk [RR] 1.20, 95% CI: 1.16–1.25) [11]. Higher BMI is also associated with increased risk of all major UI subtypes, including stress, urge, and mixed incontinence, with the strongest association seen for mixed and severe incontinence [52,53,54,55].

In postmenopausal women, those with higher BMI not only have greater prevalence but also greater severity and persistence of UI symptoms over time [56,57,58,59,60]. Weight management throughout adult life is associated with lower risk and severity of UI in later years [56].

Urinary incontinence is more common and more severe in postmenopausal women as BMI increases, with a clear dose–response relationship across the BMI spectrum [52,58,60].

#### 3.2.3. Daytime Versus Nocturnal Urinary Incontinence

In postmenopausal women, the distribution of urinary incontinence episodes is typically higher during the day than at night, but nocturnal episodes (nocturia and nocturnal incontinence) are common and increase with age and menopausal status. Nocturia—waking at night to void—is reported in approximately 25% of early postmenopausal women, with prevalence and bother increasing with the number of nocturnal voids. Women with two or more nighttime voids are more likely to report urgency and sleep disturbances, and nocturia is multifactorial, involving both bladder dysfunction and sleep disorders related to estrogen deficiency [61,62,63].

Daytime incontinence episodes, particularly stress and urgency incontinence, remain more frequent overall, but nocturnal symptoms become increasingly prominent after menopause. The prevalence of nocturia (≥2 voids/night) rises with menopausal stage, and is often associated with overactive bladder symptoms and other menopausal complaints, such as vasomotor symptoms and insomnia [64]. Bladder diaries in postmenopausal women show that maximum voided volumes are lower at night compared to the day, and nocturnal polyuria is more common in those with frequent nocturia [65].

Urinary incontinence episodes in postmenopausal women are distributed across both day and night, with daytime episodes generally more frequent, but nocturnal episodes (nocturia and nocturnal incontinence) becoming increasingly prevalent and bothersome with advancing menopausal stage [62,63,65].

#### 3.2.4. Impact of Medications

Systemic estrogen therapy, alpha-adrenergic blockers, certain antihistamines, beta receptor agonists, and angiotensin II receptor blockers (ARBs) are medications commonly used in postmenopausal women that can exacerbate urinary incontinence symptoms, including both daytime and nighttime episodes.

Systemic estrogen (oral or transdermal) has been consistently associated with an increased risk of incident and prevalent urinary incontinence in postmenopausal women, while local vaginal estrogen does not share this risk and may improve symptoms [10]. Alpha-adrenergic blockers can relax the urethral sphincter, leading to worsened stress or mixed incontinence [66]. Certain antihistamines and beta receptor agonists have also been linked to increased incontinence risk, likely due to their effects on detrusor muscle or urethral tone [67]. ARBs have shown a positive association with incontinence in women, though the mechanism is less clear [67].

Diuretics, especially loop diuretics, may increase urinary frequency and urgency, but the association with incontinence is less robust in longitudinal studies [66,68]. Polypharmacy in general is associated with a higher risk of urinary symptoms in older women [68].

In summary, systemic estrogen, alpha-blockers, certain antihistamines, beta agonists, and ARBs are the most consistently implicated medications in exacerbated urinary incontinence in postmenopausal women, affecting both daytime and nighttime episodes [67,68].

Also, alpha-adrenergic blockers, diuretics, calcium channel blockers, and CNS depressants are the most relevant non-hormonal medication classes that may worsen urinary incontinence in postmenopausal women [66,68].

#### 3.2.5. Sexual Function and Coital Incontinence

Among postmenopausal women with urinary incontinence who experience urine loss during intercourse (coital incontinence), the majority have stress incontinence as the underlying type. In a large urogynecologic cohort, 64% of women with coital incontinence had urodynamic stress incontinence, 10% had mixed incontinence, and 5% had detrusor overactivity (urgency incontinence). The remaining women had other or unclassified types. Notably, the frequency of coital incontinence episodes did not significantly differ by incontinence subtype, but women with mixed incontinence reported the greatest sexual quality of life impairment and symptom distress [69].

Community-based studies confirm that stress and mixed urinary incontinence are the most common types associated with urine leakage during sexual activity in midlife and older women, with mixed incontinence conferring the highest risk for coital leakage. The prevalence of coital incontinence among sexually active women with incontinence is high, with up to 56% reporting leakage during intercourse in specialty clinic populations, and about 25% in community samples [70].

Stress incontinence is the most common type of underlying urinary leakage during intercourse in postmenopausal women, followed by mixed incontinence, and less commonly, urgency incontinence [69,70].

The most common risk factors for experiencing urinary incontinence during sexual intercourse in postmenopausal women are as follows:

- Depression: Women with depression have nearly double the odds of coital incontinence compared to those without depression [70].
- Symptomatic pelvic organ prolapse: The presence of pelvic organ prolapse is independently associated with a higher risk of urine leakage during sex [70].
- Higher frequency of sexual activity: More frequent sexual activity is associated with increased risk, likely reflecting greater opportunity for symptom occurrence [70].
- Type of incontinence: Women with stress or mixed urinary incontinence are at significantly higher risk than those with urgency incontinence [70,71,72].
- Lower maximal urethral closure pressure (MUCP): Urodynamic evidence of reduced MUCP is an independent predictor of coital incontinence, reflecting intrinsic sphincter deficiency [69,71].
- Higher body mass index (BMI): Elevated BMI is associated with increased risk, likely due to greater intra-abdominal pressure and its effects on pelvic floor support [69,72].
- Obesity: Obesity is a well-established risk factor for all types of incontinence, including coital incontinence [13].
- Menopausal symptom burden: Greater severity of menopausal symptoms is associated with higher odds of all incontinence subtypes, including those that manifest during intercourse [24].

Other factors, such as age, race/ethnicity, and parity, are less consistently associated with coital incontinence specifically, though they are established risk factors for urinary incontinence in general [10,14]. The pathophysiology is most often related to stress incontinence mechanisms, including urethral hypermobility and intrinsic sphincter deficiency [14,69,71].

#### 3.2.6. Combination Approaches

Kegel exercises, also known as pelvic floor contractions, are an effective first step in treating urinary incontinence during menopause. Studies show that up to 92% of women may notice an improvement in both stress and urge incontinence when practicing these exercises regularly [73,74,75]. Pelvic floor muscle training (PFMT), which includes Kegel exercises, is strongly recommended as the initial therapy by the International Continence Society. Systematic reviews confirm that PFMT reduces the number of leakage episodes, improves continence rates, and strengthens pelvic floor support, which can also help with some genitourinary syndrome of menopause (GSM) symptoms.

Recent evidence shows that combining PFMT (Kegel exercises) with intravaginal estriol provides superior outcomes compared to estriol alone, with significantly higher remission rates even in women with moderate and severe symptoms of atrophic vulvovaginitis [76].

Another study focusing on women with an overactive bladder and mixed urinary incontinence showed that adding Kegel exercises to pharmacologic treatment significantly improved urinary control and quality of life over six months, more so than medication alone [77].

A recent pilot study (2025) showed that balneotherapy with saline water and mud packs induces significant microvascular changes in both dermal and muscle tissues, including an increase in capillary density and angiogenesis. These effects, which persisted for at least 24 h post-treatment, suggest that balneotherapy may enhance tissue perfusion and support regenerative processes, potentially contributing to symptom relief in menopausal women [78].

Adding local vaginal estrogen therapy to PFMT tends to bring additional benefits, especially in addressing GSM-related issues such as vaginal dryness, painful intercourse, vulvar atrophy, and urinary urgency or frequency. According to the American Society for Reproductive Medicine, vaginal estrogen improves stress and urge incontinence, as well as urgency, frequency, and nocturia, and performs better than nonhormonal moisturizers in women with more severe GSM [79]. Meta-analyses further confirm that vaginal estrogen significantly reduces urinary incontinence and other lower urinary tract symptoms, with pooled results showing meaningful improvements after treatment [80]. Unlike PFMT, vaginal estrogen directly treats atrophic changes such as dryness, dyspareunia, and vulvar thinning [79].

Although head-to-head trials of combination therapy versus single therapy are still few, the accumulating evidence suggests that combining Kegel exercises with vaginal estrogen (or estriol) is more effective than Kegel exercises alone in menopausal women who experience both incontinence and GSM symptoms [73,76,77,78,79,80]. Vaginal estrogen is usually prescribed in low-dose forms—such as estradiol tablets (10 μg twice weekly), creams (0.5 mg daily), or vaginal rings (7.5 μg daily)—with minimal systemic absorption [79,80,81].

##### 3.2.7. Surgical Treatment

Surgical management of urinary incontinence in women is determined by the incontinence subtype, with most procedures targeting stress urinary incontinence (SUI). The midurethral sling (MUS)—via the retropubic or transobturator route—is the most widely performed and best-studied option. As a minimally invasive outpatient procedure, it shows high effectiveness, with cure rates of 62–98% at one year and 43–92% beyond five years, alongside a relatively low complication profile [14,15,82,83]. The Women’s Preventive Services Initiative identifies synthetic midurethral mesh slings as the most common primary surgical treatment for SUI [83].

Between approaches, retropubic slings may yield slightly higher cure rates but carry greater risks of voiding dysfunction and bladder perforation, whereas transobturator slings are associated with less perforation but more groin pain; both are appropriate for women without significant comorbidities or prior mesh complications [14,84,85].

Other effective options include pubovaginal slings using autologous fascia, retropubic colposuspension (e.g., Burch), and urethral bulking injections. Pubovaginal slings and colposuspension are typically reserved for women who cannot undergo mesh procedures, those who have recurrent SUI after prior surgery, or present with a fixed, nonmobile urethra. Urethral bulking is less durable but minimally invasive and useful for patients with substantial comorbidities or anesthesia risk [86,87,88].

For urgency urinary incontinence (UUI) refractory to conservative and pharmacologic therapy, intravesical onabotulinumtoxinA (typically 100 U), sacral neuromodulation, and percutaneous tibial nerve stimulation (PTNS) are established options [83,87].

Adverse effects are procedure-specific. For MUS, retropubic slings are linked with bladder perforation (up to 10%), voiding dysfunction/retention (up to 10%), and suprapubic pain (up to 4%); transobturator slings show lower perforation rates (≤1%) and voiding dysfunction but more groin pain (up to 6.5%). Both approaches carry risks of mesh exposure/erosion (up to 4%), de novo urgency/urge incontinence (up to 9.5%), and, rarely, vascular or bowel injury [14,15,82,89]. Pubovaginal slings may lead to voiding dysfunction/retention (up to 15%), de novo urgency (up to 8.6%), suture exposure (up to 5.4%), and occasional wound infection; risks increase with prior pelvic surgery or poor tissue quality [86]. Retropubic colposuspension may cause voiding dysfunction (up to 11%), de novo urgency (up to 11%), suture exposure (up to 7.8%), wound infection (up to 7%), and, less commonly, bowel injury (up to 3.1%) [84,85]. Urethral bulking typically causes mild, transient effects—retention, dysuria, hematuria, or UTI—with rare serious complications [87,88]. OnabotulinumtoxinA is associated with urinary retention (8–10%) and UTI (up to 35%), with higher retention risk in older women and those with baseline voiding dysfunction [87]. Sacral neuromodulation can involve implant-site pain, lead migration, infection, or device malfunction; revisions may be required [87]. PTNS has minimal adverse effects, most commonly transient local pain or bruising (up to 8.5%), and no serious events reported [87].

Contraindications also guide selection. MUS procedures are contraindicated in active UTI, untreated urethral or vaginal pathology, poor tissue quality (e.g., post-pelvic radiation), or prior synthetic mesh complications; extensive prior pelvic surgery is not absolute but increases risk in scarred or distorted anatomy [14,15,82]. Pubovaginal slings are relatively contraindicated with impaired wound healing (uncontrolled diabetes, immunosuppression), severe obesity, or inability to tolerate general anesthesia; prior abdominal surgery may complicate fascial harvest, and dense pelvic scarring raises complication risk [86]. Retropubic colposuspension is contraindicated with significant pelvic adhesions, prior extensive retropubic surgery, or inability to tolerate abdominal surgery, and is less suitable in poor overall surgical candidates [84]. Urethral bulking is contraindicated with active UTI, urethral strictures, or marked urethral mobility, but is preferred in patients unfit for more invasive procedures [88]. OnabotulinumtoxinA is contraindicated in women with prior urinary retention, neuromuscular disorders (e.g., myasthenia gravis), or recurrent UTIs; caution is needed with impaired bladder emptying [87]. Sacral neuromodulation is avoided in active infection, anatomic constraints preventing lead placement, or inability to undergo implantation; relative contraindications include bleeding diatheses and inability to manage the device [87]. PTNS is avoided in patients with implantable electrical devices (e.g., pacemakers), infection at the stimulation site, or inability to adhere to repeated visits [87].

In practice, individualization is key: the incontinence subtype, comorbidities, prior surgeries, risk tolerance, and patient preference should drive the choice. Shared decision-making—covering realistic benefits, risks, durability, and follow-up—remains central to high-quality care.

## 4. Future Perspectives

The therapeutic landscape for urinary incontinence in menopausal women is shifting toward an increasingly individualized and multimodal approach. While conservative strategies such as pelvic floor muscle training (PFMT), laser therapy or local injection of bulking agents, electric stimulation, and local vaginal estrogen supplementation remain the cornerstone of conservative treatment, accumulating evidence highlights the superior efficacy of combination regimens in improving both continence and genitourinary syndrome of menopause (GSM)-related symptoms [73,76,77,78,79,80]. Adjunctive modalities such as balneotherapy and emerging energy-based interventions (e.g., lasers, radiofrequency) show promise in enhancing tissue vascularization, regeneration, and symptom relief, though high-quality long-term studies are still required to establish their place in routine practice [78].

For women with stress urinary incontinence (SUI) refractory to conservative or pharmacological measures, surgical management remains an essential component of care. Midurethral slings (MUS), via retropubic or transobturator route, continue to represent the most widely performed and well-validated option, with sustained cure rates and a favorable safety profile [14,15,82,83]. Alternative surgical strategies—including pubovaginal slings using autologous fascia, retropubic colposuspension, and urethral bulking injections—provide valuable alternatives in selected cases, particularly for women with contraindications to synthetic mesh or recurrent SUI [84,85,86,87,88].

For urgency urinary incontinence (UUI) resistant to first-line therapies, minimally invasive interventions such as intravesical onabotulinumtoxinA, sacral neuromodulation, and percutaneous tibial nerve stimulation (PTNS) expand the treatment armamentarium, with demonstrated efficacy but distinct adverse event profiles [83,87].

Moving forward, research priorities include refining patient selection criteria for surgical and minimally invasive therapies, optimizing multimodal regimens, and developing standardized algorithms that integrate conservative, hormonal, and surgical interventions. Emphasis should also be placed on long-term outcomes, durability of treatment, and strategies to minimize procedure-specific complications, particularly those associated with mesh use. Importantly, patient-centered approaches that incorporate shared decision-making, comorbidity assessments, and quality-of-life metrics must remain central to clinical practice [83,88,90,91].

In postmenopausal women, it is difficult to accurately classify involuntary urine loss into one of the following three categories: SUI, UUI, or MUI. We preferred to refer to the condition generically as involuntary urine loss in menopause in the context of GSM. The difficulty in definitively separating symptoms is very high, because atrophy of the vulvovaginal mucosa, pelvic muscle weakening, vaginal dryness, and involuntary urine loss cannot practically be separated when discussing sexual quality of life in postmenopausal women. This complex symptom picture is substantially reduced after Kegel exercises and local estrogen therapy, though it is difficult to specify whether the improvement in sexual quality of life is solely due to the patient losing less urine during sexual activity, or also due to the discomfort caused by insufficient vaginal lubrication, accompanied by increased friability of the vulvovaginal mucosa. It is more than evident that all these changes occur in the context of significant estrogen decline. At the same time, sexual activity decreases or even sometimes ceases in older women. These individuals are sometimes prone to a sedentary lifestyle, in which physical effort is substantially reduced. By analyzing this evidence, we wish to emphasize the importance of physical activity and perineal rehabilitation exercises, whether or not they are combined with local estrogen supplementation in these individuals. On the other hand, there are cases in which these conservative therapies are bypassed, with patients being directly offered surgical interventions, especially for correcting the urethrovesical junction. Besides the fact that these interventions can sometimes be quite aggressive for a woman in menopause, they are performed on an atrophic, low-estrogen tissue, which decreases their effectiveness and increases the number of complications, such as prosthetic material rejection or vaginal tissue erosions, with exposure of the prosthetic material. Approaching the condition through conservative therapy, even if it does not completely resolve involuntary urine loss, can create favorable conditions for subsequent surgical interventions [92]. Even though there are currently few studies in the specialized literature that demonstrate the beneficial effect of estrogen therapy on postoperative outcomes for involuntary urine loss [92], we hope that future studies will bring new evidence regarding the integration of these concepts into new therapeutic protocols.

Laser therapy, both intravaginal and intraurethral, or the intraurethral injection of bulking agents, appear to have beneficial effects in menopausal patients with SUI. They can be administered alone, together, or combined with Kegel exercises, electrostimulation, or pre- or post-surgical intervention [93,94].

### Therapeutic Limits

As for the limitations of estrogen therapy in menopause, cases of endometrial hyperplasia, or even breast or endometrial cancer, may be considered. Additionally, adverse reactions to the excipients in the products can sometimes be considerable. Kegel exercises can sometimes be performed incorrectly, both in terms of the contraction–relaxation duration and the number of repetitions The attending physician must ensure that the patient has fully understood the protocol for performing Kegel exercises [76,77]. Laser therapy may represent an alternative or perhaps a complement to conservative therapies. Both laser therapy and electrostimulation are not accessible to all patients, often due to economic reasons, especially for patients with a low socio-economic status in countries where national health insurance does not financially cover these procedures. Additionally, transportation to the clinics where these procedures are carried out can be a problem, especially for elderly or less mobile patients.

Surgical interventions can also be burdened by numerous limitations, both in terms of the presence or absence of surgeons with expertise in urogynecology, a lack of prosthetic materials, long patient waiting lists, associated comorbidities that make surgery inadvisable, extremely atrophic vulvo-vaginal tissues, or mesh rejection, etc. [93].

Some meta-analyses make various observations regarding the surgical complications of cases of involuntary urine loss, whether or not associated with genital prolapse.

Erosions, wound granulation, and dyspareunia may occur after vaginal prolapse repair with graft materials, though rates vary widely across studies [95].

While transvaginal permanent mesh is associated with lower rates of awareness of prolapse, repeat surgery for prolapse, and prolapse on examination than native tissue repair, it is also associated with higher rates of total repeat surgery (for prolapse, stress urinary incontinence, or mesh exposure), bladder injury, and de novo stress urinary incontinence [96].

In fact, involuntary urine loss during menopause and the success of its surgical treatment depend on a combination of conditions, including age, genetic factors, lifestyle, and obstetric history, as well as the ability of the ligamentous apparatus to recover after pregnancy and childbirth.

Vaginal delivery, parity, birthweight, age, body mass index, levator defect, and levator hiatal area are risk factors, and cesarean delivery and smoking are protective factors for primary prolapse. Preoperative prolapse stage and younger age are risk factors for prolapse recurrence after native tissue surgery [97].

Another limitation of therapies for involuntary urine loss is elderly, frail, and malnourished patients. In these cases, malnutrition can lead to serious deficiencies in ligament or muscle structure. These elderly and malnourished individuals may also be affected by major depressive syndromes [98].

## 5. Conclusions

Urinary incontinence in menopausal women represents a multifactorial condition with a significant impact on quality of life. Current evidence supports a stepwise, individualized therapeutic approach, starting with conservative strategies such as pelvic floor muscle training and electric stimulation and progressing to local hormonal therapy, minimally invasive interventions, or surgical management depending on symptom severity, subtype of incontinence, and patient characteristics.

Combination therapies—particularly the integration of pelvic floor rehabilitation with local estrogen supplementation—can offer superior efficacy in urinary incontinence in menopause. Surgical and neuromodulatory options remain indispensable for refractory cases, provided that patient selection and risk stratification are carefully performed.

Future directions emphasize the refinement of multimodal algorithms, the durability of outcomes, and the incorporation of patient-centered outcomes into clinical decision-making. Ultimately, optimal management requires tailoring interventions to individual needs while balancing effectiveness, safety, and quality of life.

## Data Availability

No new data were created or analyzed in this study. Data sharing is not applicable to this article.

## Abbreviations

The following abbreviations are used in this manuscript:

UI	urinary incontinence
SUI	stress urinary incontinence
UUI	urgency urinary incontinence
MUI	mixed urinary incontinence
GSM	genitourinary syndrome of menopause
PTNS	percutaneous tibial nerve stimulation
